# Saliency Map and Deep Learning in Binary Classification of Brain Tumours

**DOI:** 10.3390/s23094543

**Published:** 2023-05-07

**Authors:** Wojciech Chmiel, Joanna Kwiecień, Kacper Motyka

**Affiliations:** Faculty of Electrical Engineering, Automatics, Computer Science and Biomedical Engineering, Al. Mickiewicza 30, AGH University of Science and Technology, 30-059 Krakow, Poland; wch@agh.edu.pl (W.C.); kmotyka@student.agh.edu.pl (K.M.)

**Keywords:** deep learning, convolutional neural networks, CAM, Grad-CAM, binary brain tumour classification

## Abstract

The paper was devoted to the application of saliency analysis methods in the performance analysis of deep neural networks used for the binary classification of brain tumours. We have presented the basic issues related to deep learning techniques. A significant challenge in using deep learning methods is the ability to explain the decision-making process of the network. To ensure accurate results, the deep network being used must undergo extensive training to produce high-quality predictions. There are various network architectures that differ in their properties and number of parameters. Consequently, an intriguing question is how these different networks arrive at similar or distinct decisions based on the same set of prerequisites. Therefore, three widely used deep convolutional networks have been discussed, such as VGG16, ResNet50 and EfficientNetB7, which were used as backbone models. We have customized the output layer of these pre-trained models with a softmax layer. In addition, an additional network has been described that was used to assess the saliency areas obtained. For each of the above networks, many tests have been performed using key metrics, including statistical evaluation of the impact of class activation mapping (CAM) and gradient-weighted class activation mapping (Grad-CAM) on network performance on a publicly available dataset of brain tumour X-ray images.

## 1. Introduction

Classification and detection methods that rely on deep learning approaches learn to predict classes or detect objects. These approaches have the ability to automatically extract features from the data and can achieve good results. They often do not have difficulty generalising different datasets whose characteristics may vary. The popularity of deep learning has motivated researchers to search for methods that are useful for medical applications. Some papers have focused on deep learning methods that use various images of different diseases of the brain. In this paper, we suggest using convolutional neural networks (CNNs) that can use images as input directly, combined with methods that determine saliency areas, to make a more reliable classification of brain images with and without tumours.

A brain tumour is the growth of cells within the brain in an abnormal way. The prognosis of a brain tumour depends on many factors, for example its location, its histopotology, and in general, a correct detection and diagnosis is crucial for successful treatment planning. Early detection of brain tumours is crucial and is a very important step of patient diagnosis [1]. It is extensively investigated by many researchers and performed by using, e.g., magnetic resonance image (MRI), or computed tomography. Naturally, manual inspection of such images aimed at detection of brain tumour cases is a time-consuming process, so proper automatic brain tumour diagnosis receives great attention about research on medical image processing. It is worth mentioning that achieving better accuracy in brain tumour image classification remains a challenge.

The saliency maps detect the dominant object and various parts of the background. They should include low- and high-level factors, such as colour, contrast, suppressed features, the possibility of existence of more centres of mass, etc. The idea of a saliency map is based on the behaviour of living beings that guide attention and gaze to the most conspicuous region in a visual scene. The saliency-map theory emerges and is implemented in several domains of science such as psychology, neuroscience, machine vision, defence, logistic, medical diagnosis, advertising, and diagnosis [2].

### 1.1. Related Work

Some papers have focused on different saliency methods and artificial intelligence (AI) methods in various medical applications. For example, in [3] the authors quantitatively evaluated several saliency methods (e.g., Grad-CAM) across multiple neural network architectures in the context of aid in diagnostic decision making. The authors found that in the case of chest X-ray interpretation, all saliency methods tested perform significantly worse compared to the human benchmark. Wang in [4] examined gradient-based saliency mapping on an artificial intelligence regression model to determine hand bone age from X-ray radiographs. In the proposed approach, the partial derivative (PD) of the inferred age with respect to the intensity of the input image at each pixel served as a saliency marker to find sensitive areas contributing to the outcome. Ghosh et al. in [5] studied the interaction of robots working in 3D space and proposed a bio-inspired bottom-up attention model that takes advantage of event-driven sensing to generate depth-based saliency maps that allow a robot to interact with complex visual input. Amorim et al. in [6] proposed an approach to evaluate the faithfulness of the saliency maps by introducing natural perturbations in the image, based on the substitution of the oppose class. The authors studied their impact on evaluation metrics adapted from saliency models using a breast cancer metastases detection dataset. The results presented showed that Grad-CAM, Guided-GradCAM, and gradient-based saliency map methods are sensitive to natural perturbations and correlate with the presence of tumour evidence in the image. Ayhan et al. [7] analysed three different network architectures and developed ensembles of DNNs to detect diabetic retinopathy and neovascular age-related macular degeneration from retinal fundus images and optical coherence tomography scans. The results were validated on the basis of a direct comparison of saliency maps with the expert annotations of disease-specific pathologies and perturbation analyses using also expert annotations as saliency maps.

Some studies have focused on the applicability of methods based on the use of machine learning and deep learning techniques for the classification of brain tumours. For example, Saeedi et al. in [8] proposed AI methods to classify three types of brain tumours. They developed a 2D CNN, a convolutional auto-encoder network, and six common machine learning techniques. In [9], the pre-trained EfficientNetB0 architecture, for brain tumour classification, was applied with transfer learning. The dilated U-Net-based CNN model was introduced in [10]. Khan et al. [11] proposed an automated multimodal classification method using two pre-trained convolutional neural network models (VGG16 and VGG19), for the classification of types of brain tumours. In turn, Amin et al. [12] integrated a convolutional neural network with a discrete wavelet transform used for the fusion process to obtain more information about the tumour region compared to a single sequence of MRI, to better differentiate tumour and non-tumour regions. Sajjad et al. in [13] proposed multi-grade brain tumour classification system with a CNN, in which a pre-trained CNN model was tuned with data augmentation. In [14], the basic inception residual network (Inception-ResNet-v2) and the deep dense network were employed for three-class brain tumour classification. This model had three dense layers before the softmax layer. In turn, in [15], a hybrid approach based on convolutional neural network and long short-term memory (CNN-LSTM) for classifying brain tumour was presented.

### 1.2. Contribution

Our contribution addresses the demonstration of the usefulness of various CNNs with saliency areas to detect brain tumours in images, and is an attempt to answer a question of how these networks take similar or distinct decisions based on the same set of prerequisites.

The primary aim of this paper is to examine the reasoning behind the predictions made by deep neural networks. Although a deep network may produce high-quality predictions, it is not always an indication that the prediction was based on accurate assumptions. In contrast to other machine learning methods, where knowledge is typically represented in a human-readable format, deep learning knowledge is gathered in the form of neurone weights, which are often challenging for humans to interpret.

With the wide array of neural network architectures available today, it is crucial to understand their decision-making processes. Furthermore, understanding the discrepancies in knowledge processing between various networks is essential, such as where the network concentrates its attention on the analysed image when making decisions. An additional consideration is to elucidate the influence of knowledge transfer on saliency maps. A feasible approach to achieve this is to compare the resultant saliency maps in the two scenarios. Another significant concern is the quality of the dataset used for training. If the dataset is of low-quality, the resulting trained network may arrive at incorrect decisions or base its decisions on flawed assumptions.

Therefore, we would like to provide an insight into the issues that occur while using these methods. In this paper, we focus only on the binary brain tumour image classification known as brain pathology detection, which classifies the brain image as normal or abnormal. This approach is especially useful during population screening. Although there are papers on the topics of saliency maps, deep learning methods, and the pathological brain, we have found none that covers all three of these topics. For that reason, our experiments consist of testing four CNNs on a publicly available dataset of X-ray images [16] for brain tumour detection. We use three pre-trained VGG16, ResNet50, EfficientNetB7 networks and our own convolutional network. For each of the above networks, to find regions of an image that were influential to the classification task, we include the CAM and Grad-CAM methods.

It should be mentioned, that the usage of various pre-trained CNNs for computer vision tasks have been described in some papers. For example, in [17], deep learning architecture based on the decision fusion of image quality coming from six types of CNNs has been presented.

### 1.3. Framework of Research

**Figure 1:**
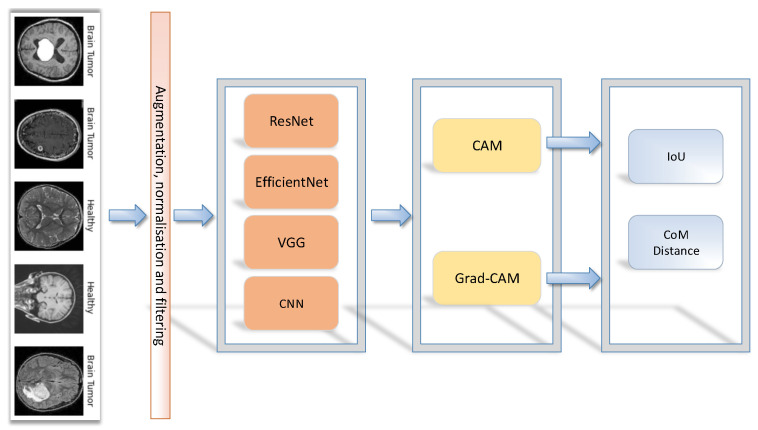
Evaluation framework of saliency methods.

Our methodology is shown in Figure 1, where the outcome represents the performance of the model during binary brain tumour classification. An image was fed into the network after normalisation. Furthermore, data augmentation was used before the training process, to balance the image dataset by making image modifications. To compare the CAM and Grad-CAM methods, the performance was calculated using the intersection over Union (IoU) and the centre of mass (CoM). For the VGG16, ResNet50, EfficientNetB7 architectures, the transfer learning technique was used, in which pre-trained models (with the last modified layers) were again trained with new data to obtain a better performance of the models to solve similar tasks.

### 1.4. Structure

The rest of the paper is given as follows: Section 2 is devoted to the discussion of existing deep network architectures used as backbone models for image analysis, and the analysis of saliency areas as methods of interpreting the results of deep neural networks. Section 3 explains the popular indicators used to perform statistical comparisons of saliency map analysis and describes the results obtained, including the impact of the CAM and Grad-CAM methods. Concluding remarks and future work are given in Section 4.

## 2. Methods

### 2.1. Architectures of Deep Neural Networks

Intensive studies on deep learning methods have produced several neural network models in recent years. The purpose of CNNs is to extract higher-order features from data by convolution. These networks are great for recognising objects in images, which was one of the main reasons why the world appreciated the power of deep learning. CNNs prove to be most useful in problems where the input data have a specific structure (images or sounds). Values in this type of data create spatial relationships.

There are many varieties of CNNs that differ in layer arrangement. There are generally three main groups of layers:

- input layer, which processes the spatial data of the image;
- feature-extracting layers—they are arranged in a general sequence containing a convolutional layer that uses numerous filters to learn various features of data received from the input layer (the obtained result is transformed using the ReLU activation function), and a pooling layer (the task is to gradually reduce the spatial size of the data representation);
- classification layers or output layer (in most cases, it is a fully connected layer) used to compute class scores as a result of network operation.

There are many convolutional networks; it is impossible to discuss all of them. Therefore, we will limit ourselves to a brief presentation of the VGG16, ResNET50, and EfficientNetB7 architectures used in our research as backbone models. For these three selected ready-made network architectures, transfer learning was used to obtain high-precision models for our problem. In addition, our simple convolutional neural network is used for the evaluation of the network performance for the problem considered without using transfer learning, as in previous architectures.

The first model considered, the VGG architecture, was defined by the Visual Geometry Group, who demonstrated that network depth is a critical factor in network performance [18]. It had, depending on the version, 16 to 19 weight layers and worked in such a way that in subsequent layers the resolution of the input image was reduced, while the number of filters used was increased. VGG16 has thirteen convolutional layers, five maximum pooling layers, and three dense layers. It consists of 21 layers, but only has 16 layers with learnable parameters. Moreover, this model has convolution layers of a 3 × 3 filter with stride 1, max pooling layers of a 2 × 2 pool size with stride 2. It is worth adding that in the case of convolutional layers, the only activation function used by the authors was ReLU, and after all layers, three fully connected layers were used. The first two layers consisted of 4096 neurones, and the last one, due to the problem being solved, contained 1000 neurones and the softmax activation function.

The Residual Network (ResNet) model [19] uses “skip connections” to solve the vanishing gradient problem, allowing inputs to skip some convolutional layers. ResNet50 denotes the architecture that can work with 50 layers. An important part of the ResNet architecture includes so-called residual blocks. They introduce the addition of the input to the output of a series of convolution blocks. The ResNet network is built in such a way that residual connections occur every few layers. Those networks become more effective with each added layer (until they are over-trained), and the more layers the network has, the more residual connections have a positive effect on its performance. For the ResNet networks that have more than 34 layers, special structures (three-layer bottleneck blocks) consisting of three layers are used: 1 × 1, 3 × 3, and 1 × 1 convolutions. Adding more convolutional layers per block, widening the convolutional layers, or increasing their filter sizes might increase the representational power of residual blocks.

The EffcientNet model proposed in [20] uses a method that uniformly scales all three dimensions (depth, width, and resolution) using an effective compound coefficient. It was proven that scaling in three dimensions simultaneously gives the best results of the overall performance of the model considering the mutable available resources compared to scaling in single dimensions. The network is fine-tuned to obtain maximum accuracy. Moreover, the network is penalised for a slow inference time and if it is very computationally heavy. In order to significantly improve network performance, the baseline architecture must also be appropriate. When creating the EfficientNet network, their authors defined a base architecture mainly consisting of a mobile inverted bottleneck. The EfficientNet contains models from B0 to B7. In the presented experiments EfficientNetB7 is used as a backbone model.

The last network, simply named CNN, is our model created. It is less complicated, and it can be treated as an example that confirms the need to use transfer learning. This network consists of four convolutional layers, where some of which are pooling layers (with max pooling operations). The first convolutional layer has 16 filters of size 2 × 2, all subsequent layers have filters of size 3 × 3. In addition, as the depth of the network increases, the number of filters increases to allow more abstract features to be captured. As mentioned, the first layer has 16 filters; the next ones have 32, 64, 128, respectively. The ReLU ativation function was used. All these layers are followed by a global averaging pooling. Moreover, the softmax activation function was added to the output layer (as a classification layer).

### 2.2. Saliency Maps

Saliency maps give better insight into the decision making of neural networks. They contain information on recognising objects from the background of images and might help to understand what the individual convolutional layers focus on. Methods of determining saliency areas can help you assess which networks have performed well. These areas can show that some networks focused on the wrong parts of the image. Because ground truth segmentations for medical imaging are time consuming, expensive to obtain, and generally not accessible, we decide to make binary comparisons between different network models and saliency maps.

#### 2.2.1. Class Activation Mapping

Convolutional networks can act as object detectors. If the last fully connected layer of neurones is replaced by a global average pooling (GAP), the quality of the achieved object location will be very high. The class activation mapping (CAM) method [21] can be used to create saliency areas, and is described as the summation of the dot product of the last convolutional feature maps and the class-wise weights of the fully connected layer (applied after global average pooling). This method is distinguished by the fact that localisation objects can be obtained in a single forward propagation of the network, but the results are very general. In order to use the CAM method, the neural network must mainly consist of convolutional layers and just before the output layer it should perform the GAP operation on the feature maps, and use the result of this operation as the input to the fully connected layer. With this linkage structure, we can identify the influence of given image regions on a decision by backprojecting the output layer weights onto convolutional feature maps. As a result, GAP returns the spatial average of the feature map of each neurone in the last layer of the network, and the weighted sum of these values is used to calculate the network performance. To calculate CAM we perform similar calculations. Therefore, in the case of image classification, this process can be described as follows: Let $f_{k}\left(x,y\right)$ be the activation value of the neurone *k* of the output layer for the value at position (*x*, *y*) in the feature map. Then, for neurone *k*, the result of global joining averaging is as follows:

(1) $$F^{k}=\sum_{x,y}f_{k}\left(x,y\right)$$

For the class *c*, the input to the classifier softmax can be given as:

(2) $$S_{c}=\sum_{x,y}\sum_{k}w_{k}^{c}f_{k}\left(x,y\right)$$

where $w_{k}^{c}$ denotes the weight of the $F_{k}$ value for the class *c*. Therefore, the activity map $M_{c}$ for class *c* can be defined as follows:

(3) $$M_{c}\left(x,y\right)=\sum_{k}w_{k}^{c}f_{k}\left(x,y\right)$$

As one can see $S_{c}=\sum_{x,y}M_{c}\left(x,y\right)$, so the class activation map directly indicates the weight of a given activation in the spatial grid of pixels (*x*, *y*) leading to the classification of the image into a given class. As with other methods, units should be activated by certain visual shapes, so $f_{k}$ is a map of the occurrence of shapes in the image. In this case, the class activation map is a weighted linear sum of the occurrence of successive shapes in different places in the image space. This means that by extending the class activation map to the size of the input image, we are able to identify which part of the image had the greatest impact on decision making. Therefore, the above method could be used as a saliency map to assess the performance of deep neural networks.

#### 2.2.2. Grad-CAM Method

The Grad-CAM [22] method uses a gradient to combine feature maps, so it does not require changes in the network architecture and can be used for a wide variety of CNN models. This method uses the gradient information flowing into the last convolutional layer to assign weight values to each neurone for a particular decision. According to [22], Grad-CAM is a generalisation of the CAM method for more convolutional network architectures. The CAM method creates a saliency map for a convolutional network for image classification if we add a global average pooling layer before the softmax layer. The Grad-CAM method obtains the location of important regions in the image during one forward propagation and partial backpropagation for each image.

Given the input image and the category of interest as the output, we propagate forward through the convolutional part of the network and then through the task-specific part of the network to obtain the result for the category. The gradients of all other classes are set to 0, and the classes of interest to 1. Then the gradient signal is backpropagated to the convolutional feature maps that we are interested in and want to combine to compute the resulting saliency map.

As shown in [22], to obtain a $L_{Grad-CAM}^{C}$ saliency map with width and height for class *c*, we first calculate the gradient of the result of the last layer of the network before softmax $y^{c}$ with respect to feature map activation $A^{k}$ of the convolutional layer. In this way, we obtain multidimensional gradients with the size of feature maps. These gradients flowing back are global averaged pooled over two dimensions: width *i* and height *j*, to calculate the neurone weight $\alpha_{k}^{c}$, which represents a partial linearisation of the deep network downstream from *A*:

(4) $$\alpha_{k}^{c}=\frac{1}{Z}\sum_{i}\sum_{j}\frac{\partial y^{c}}{\partial A_{ij}^{k}}$$

Then, we multiply the weights $\alpha_{k}^{c}$ with the corresponding feature maps $A^{k}$, we sum up the components of the final saliency map, and follow this weighted combination by the ReLU function to obtain:

(5) $$L_{Grad-CAM}^{C}=ReLU\left(\sum_{k}\alpha_{k}^{c}A^{k}\right)$$

## 3. Results of Experiments and Discussion

**Figure 2:**
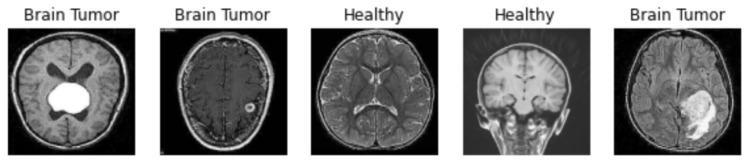
Brain image examples.

**Figure 3:**
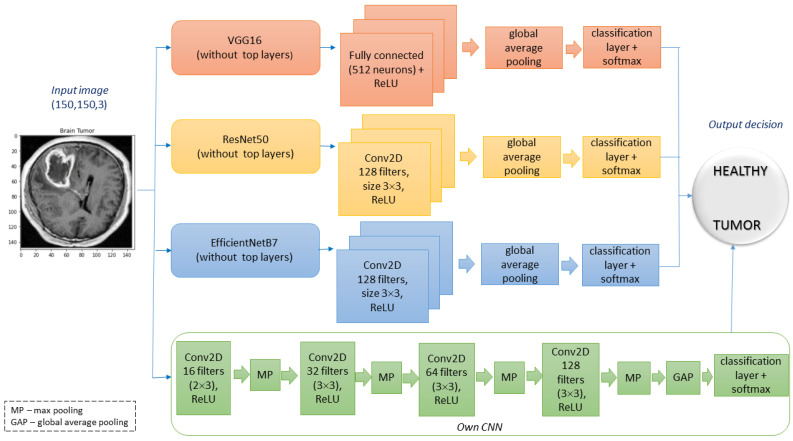
Scheme of the architectures used in the research.

To test the performance of saliency areas, we decided to choose one of the most popular types of medical datasets available on the Kaggle community platform [16], which contains 4600 X-ray images of brains with labels and separate features. Figure 2 shows brain images samples for two classes (tumour and healthy). For quality analysis, we compare the ability of state-of-the-art saliency maps in classifying images of brain tumours on three deep learning networks, i.e., VGG16, ResNet50, EffcientNetB7, and own model CNN (see Figure 3).

Pictures of sick people make up about 55% of the total images. The dataset had to be divided into two sets: 80% of all images were included in the training set, and 20% in the testing set. In the first stage, data preprocessing was performed. The images have been scaled to size 150 × 150 × 3. It was also necessary to apply data augmentation consisting of increasing the number of input images through appropriate rotations, zooming in, and changing the sharpness.

The upper layers of the models were omitted in order to apply transfer learning. The results obtained for the architectures in this way were compared with the results for a fairly simple convolutional network consisting of a few layers. The parameters of all layers of the base models (VGG16, ResNet50, EfficientNetB7) were treated as constant, because research showed that such models were able to achieve much better accuracy.

Based on our preliminary research (aimed at determining various areas of input images, e.g., cross-sectional brain, covering the neck and skull, covering the eye sockets, normal brain) to compare the methods that determine the saliency areas, we decided to limit our considerations on the tests of the CAM and Grad-CAM methods.

The statistical comparison of the saliency maps obtained using the CAM and Grad-CAM methods involved the use of two metrics described below.

### 3.1. Metrics

There are various metrics that have been designed for saliency evaluation. Moreover, it should be mentioned, there are some quality assessment models with saliency detection, for example, the image quality evaluation metric based on saliency or the reduced-reference of point clouds via content-orientated saliency projection. For example, Zhang et al. [23] proposed a visual saliency-induced metric that assumes that the visual saliency map of an image correlates with perceptual quality. In turn in [24], the image-based reduced-reference point cloud quality assessment method via saliency projection was proposed. In our manuscript, the performance comparison of the presented approaches was conducted using popular indicators, including the intersection over union (IoU), and the centre of mass (CoM).

IoU [25] is a metric (also known as the Jaccard index) used to assess the coverage of two sets by dividing the overlap between the predicted (*A*) and ground truth (*B*) annotation by their union as follows:

(6) $$IoU=\frac{\left|A\cap B\right|}{\left|A\cup B\right|}$$

This metric was used to compare the similarity of the saliency areas determined by different methods. In order to be limited to the area where the neural network was mainly focused, binarization had to be performed first. The Otsu method was used, which allows automatic determination of the binarization threshold. A fixed binarization threshold for the problem being solved would not work correctly because it depends on the network architecture used (some of them return stronger activation of neurones) and also on the input image (a brain tumour is sometimes more visible). The binarized saliency maps were compared using the IoU metric, and then the average coverage for all test images was also calculated.

CoM is another statistical method for comparing the performance of saliency maps. For this purpose, the geometric CoM was determined for each saliency map using the following formula:

(7) $$\left\{\begin{array}{c} X_{cm}=\frac{\sum_{i=1}^{W}\sum_{j=1}^{H}x_{ij}\cdot p_{ij}}{W\cdot H}\\ Y_{cm}=\frac{\sum_{i=1}^{W}\sum_{j=1}^{H}y_{ij}\cdot p_{ij}}{W\cdot H} \end{array}\right.$$

where $X_{cm}$ and $Y_{cm}$ are coordinates *x* and *y* of the mass centre, *x* and *y* are coordinates of pixels, *H* is the image height and *W* is the image width, and $p_{ij}$ indicates the value of the pixels at position i,j.

Moreover, for the performance analysis of selected networks, several performance indicators, i.e., accuracy, precision, and recall, have been calculated based on the confusion matrix. Accuracy refers to the ratio of all correctly classified instances to the overall input instances. The recall measure (also called sensitivity) informs about the proportion of actual positives that have been correctly classified. Another metric is precision, which is the ratio of true positives to all instances classified as positives (true or false).

### 3.2. Comparison of Convolutional Networks

**Figure 4:**
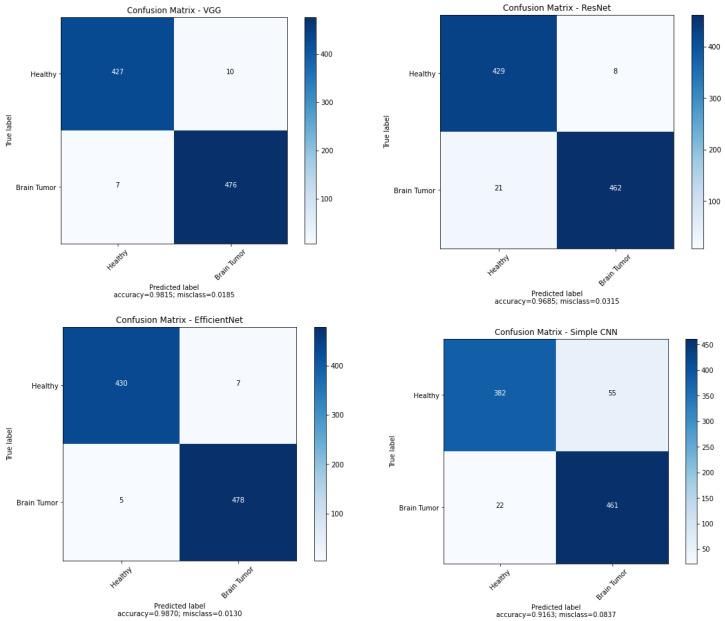
Confusion matrices for considered networks.

We developed many experiments to compare the performance of the trained networks. Confusion matrices in the context of the best results obtained for these networks are shown in Figure 4. As shown in the confusion matrix, in most cases from the testing set, VGG16 performs very well. In the case of faulty operation of the VGG16 network, a situation in which the model considers a healthy person to be sick more often than not recognising a brain tumour, so the sensitivity of the model is higher than the specificity. In the case of the ResNet50 model, sensitivity is at a lower level than specificity. This is a less desirable result because sensitivity is very important in diseases. For EfficientNetB7, the best specificity and sensitivity were obtained; for the test images this network made only 12 misdiagnoses. Our simple convolutional network makes significantly more errors compared to previous models (78 in total for the testing set), and the number of errors in the case of sick people is more than two times lower.

**Figure 5:**
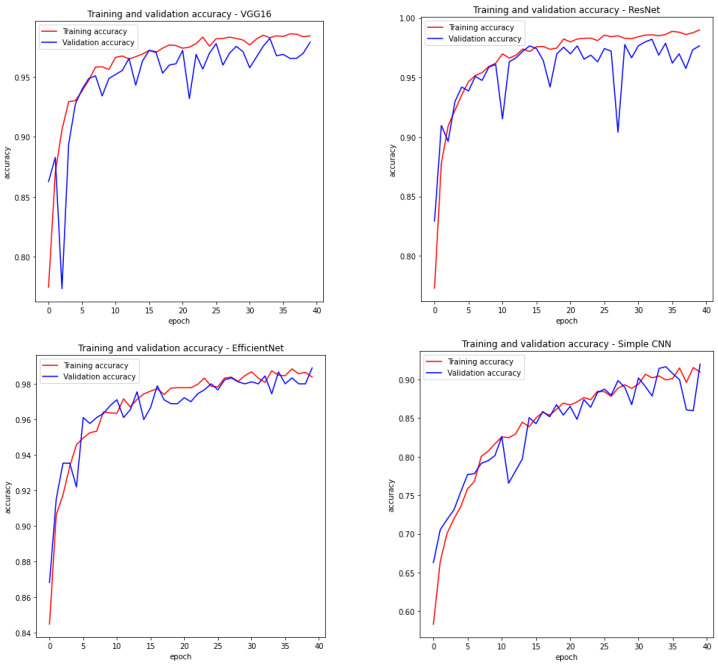
Training and validation accuracy of the proposed models.

Therefore, we have a summary describing the most important features of each network in Table 1. Moreover, Figure 5 visualises the training accuracy and testing accuracy for the considered models within 40 epochs. For three models based on backbone networks, the selected number of epochs was sufficient, because the value of model precision stopped increasing.

As you can see, the number of layers varies greatly between architectures, from 10 layers for a simple CNN network to 813 layers for a network based on the EfficientNetB7 model. It is worth noting that although the network based on EfficientNetB7 has more than 15 times more layers than the network based on ResNet50, thanks to the scaling applied in the first of the considered architectures, it has only about 2.5 times more parameters. Taking into account the number of parameters that change during training, it can be seen that EfficientNetB7 has most of them (2,949,506), and CNN has the least (97,458). The simplest network (CNN) has the worst efficiency, which is visible in the sensitivity and specificity values, which indicate the percentage of sick and healthy people correctly diagnosed. Among the trained models, there are those that have greater specificity, but also those that have better sensitivity. Generally, for medical applications and screening, a model that is more sensitive would obviously be a better choice because it is better to label a healthy person as sick than to downplay someone’s illness.

### 3.3. Results Obtained by the CAM and Grad-CAM Methods

In the case of the CAM and Grad-CAM methods, the resolution of the obtained saliency map depends on the size of the filters in the layer for which it is determined. Thus, if we compute significance maps for the topmost layers, the more convolutions and pooling operations that are performed, the lower the resolution of the resulting map.

The first method we decided to thoroughly test was CAM. Images showing brain tumours were taken for the study. In this case, the network should focus on it when making decisions, which should increase the consistency of features that affect the decisions made by the network. The conducted research confirms this and shows that the average IoU value for the CAM method for images containing a brain tumour is approximately 61.75%, and for all images this value is approximately 61.19%. In addition, in the case of images with a brain tumour, coverage for the best-performing networks, ResNet50 and EfficientNetB7, increases.

**Figure 6:**
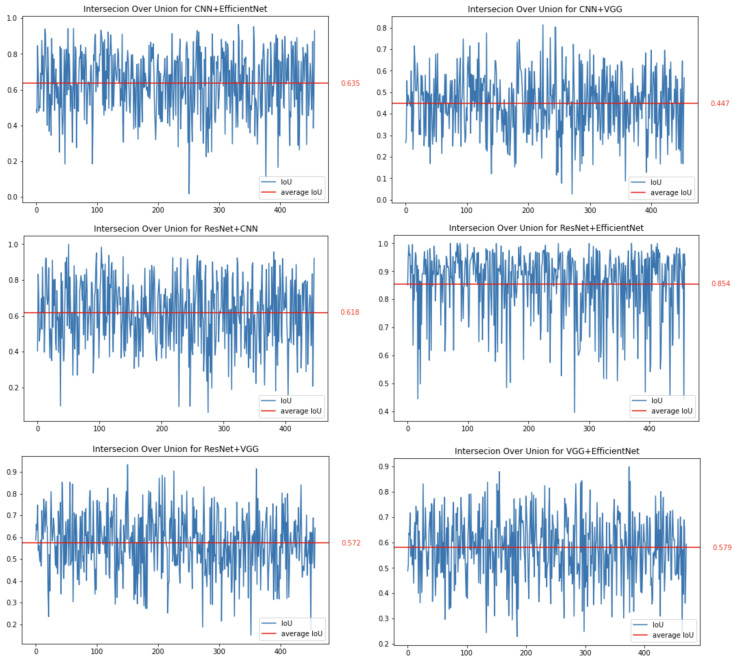
Binary comparison of IoU for all combinations of trained networks for the CAM method.

Figure 6 shows the results obtained. Each graph in this figure shows a comparison (using IoU) of the results for two different network architectures. The red horizontal line represents the average value of the IoU parameter for all analysed images. The most overlapping sets occur for the ResNet50 and EfficientNetB7 networks (the IoU parameter is set to 85.4%). The lowest coverage (with an average of 44.7%) is for the pair of the simple CNN and VGG16 networks.

**Figure 7:**
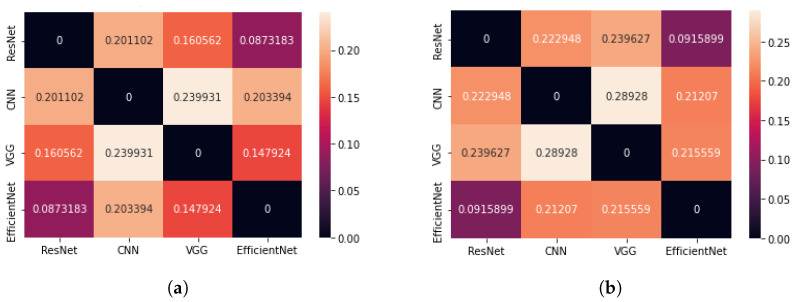
Matrix showing the average difference of Cartesian distance for four neural network architectures (the CAM method); (**a**) case: all test images, (**b**) case: all test images containing the brain tumour.

The second parameter used to evaluate the results of the CAM method for different architectures, which was based on the geometric centre of mass, also indicated the smallest difference between the centres of mass for the ResNet50 and EfficientNetB7 networks (see Figure 7). On average, it is about 8.7% of the diagonal length of an image when we analyse all images and 9.2% of the diagonal when we consider only images containing the brain tumour. Thus, the saliency areas for these two architectures are the most similar. When comparing the CoM method of the VGG16 network with the CoM of the ResNet50 and EfficientNetB7 networks, very small differences can be obtained. This may lead to the conclusion that the VGG16 network indicates a similar saliency area but consists of fewer pixels.

**Figure 8:**
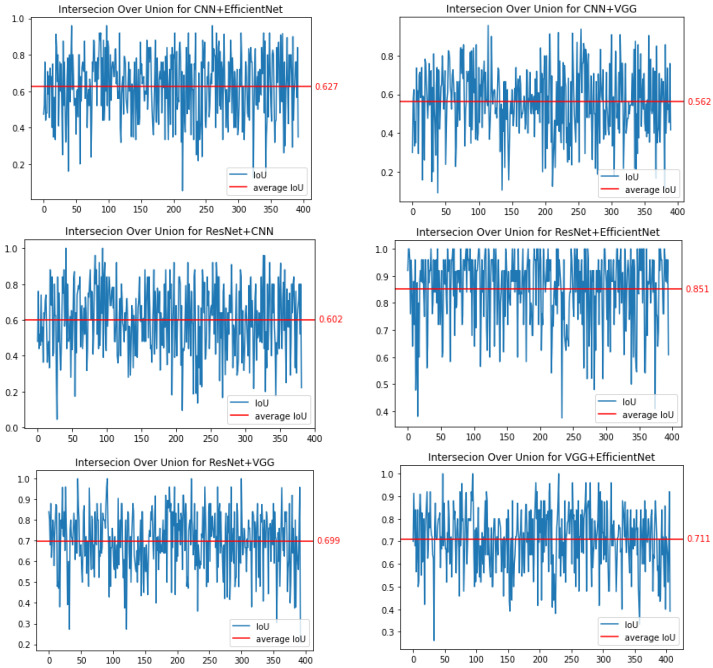
Binary comparison of IoU for all combinations of trained networks for the Grad-CAM method.

To verify the quality of the Grad-CAM method, we decided to conduct similar experiments. For this purpose, Figure 8 shows the results obtained for IoU. The highest coverage value, measured by the IoU parameter, was obtained for the combination of ResNet50 and EfficientNetB7, and it was 85.1%. For the combination of CNN and VGG16 networks, the coverage was 56.2% (note that for the CAM method it was lower). In the case of these networks, we observe very large fluctuations in the value of the IoU parameter, which may indicate the lack of stability of the saliency areas. For the CNN-ResNet50 and CNN-EfficientNetB7 pairs, the IoU factor was 60.2% and 62.7%, respectively. For the VGG16-ResNet50 and VGG16-EfficientNetB7 pairs, this parameter was equal to 69.9% and 71.1%, respectively.

**Figure 9:**
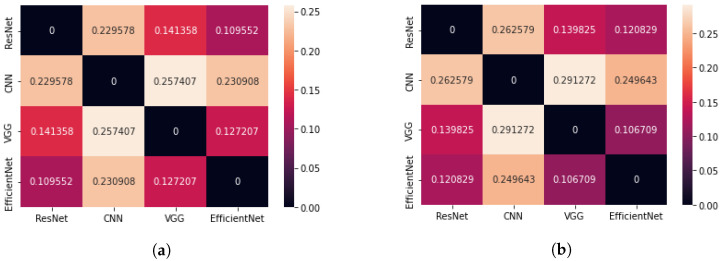
Matrix showing the average difference of Cartesian distance for four neural network architectures (Grad-CAM method); (**a**) case: all test images, regardless of whether they contain brain tumour (**b**) case: only test images that contain brain tumour.

Figure 9 shows a comparison of the average Cartesian distance for the centres of mass of the significancy maps using the Grad-CAM method. The smallest mean difference in CoM occurs for the pair of the EfficientNetB7 and VGG16 networks, as well as EfficientNetB7 and ResNet50. The largest value was obtained for the VGG16 and CNN networks. It should be noted that for pre-trained models, images with brain tumours have average CoM distances between 10.7% and 14%.

**Figure 10:**
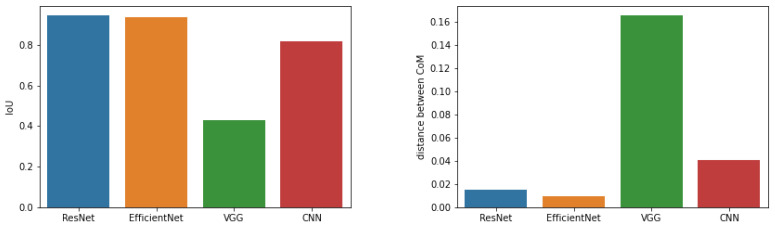
Comparison of CAM and Grad-CAM methods.

Furthermore, Figure 10 shows a comparison of the results of the CAM and Grad-CAM methods, using the average Cartesian distances between the centres of mass, as well as the IoU parameter. In the case of ResNet50 and EfficientNetB7 networks, the coverage measured by the IoU parameter is the highest (approx. 94.5%), and the average Cartesian distance of CoM is approximately 1.5% and 1%, respectively. In the case of the own simple CNN network, the IoU parameters are quite high (over 81.9%), but the difference in CoM is about 4.1%. The VGG16 network looks the worst, the IoU factor is very low (about 42.8%) and the difference in CoM is greater than 16.6%. The results obtained by the Grad-CAM method, especially for the VGG16 network, have a much smaller area (small coverage), and their centres of mass are slightly shifted.

**Figure 11:**
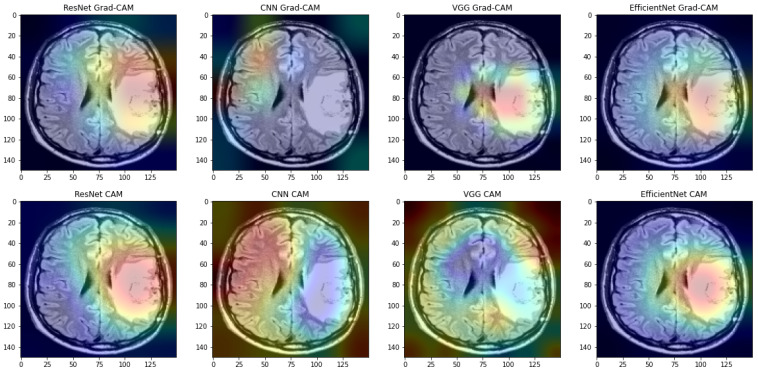
Examples of saliency maps obtained by CAM and Grad-CAM.

In some cases, incorrect operation of the Grad-CAM method has also been observed. If the image of the skull was taken from the back or side and includes more fragments than just the brain, the Grad-CAM method very often does not return the correct result. However, in cases where Grad-CAM works correctly, it almost always returns a smaller, more specific saliency area than the CAM method (see Figure 11).

To facilitate analysis of the CAM and Grad-CAM methods, the results of all the tests are summarised (case: only test images containing brain tumour) in Table 2, where:

- $E_{CaD}=\frac{CaD_{CAM}-CaD_{GradCAM}}{CaD_{CAM}}\cdot 100\%$ denotes the percentage average value of the difference between the average Cartesian distance of CoM for the CAM and Grad-CAM methods,
- $E_{IoU}=\frac{IoU_{CAM}-IoU_{GradCAM}}{IoU_{CAM}}\cdot 100\%$ denotes the percentage average value of the difference between the average IoU value for the CAM and Grad-CAM methods.

In the case of the pair ResNet50 + EfficientNetB7, both methods achieve identical outcomes. However, an interesting finding is that the Cartesian distance (CaD) between the centres of gravity was one of the highest. This suggests that the CAM method outperforms the Grad-CAM method, as evidenced by the significantly smaller Cartesian distance in the former, resulting in more stable areas. In summary, the CAM method produced superior results to the Grad-CAM method.

When comparing CNN and VGG16 networks, it is noteworthy that the difference between the IoU is substantial, reaching up to 25%. Additionally, the Cartesian distance is also significant. Moreover, the difference in the CaD parameter value for both methods is marginal, indicating that both networks exhibit a wide spread of areas, thereby rendering the resulting areas unstable. Consequently, it can be inferred that both networks may base their decisions on incorrect assumptions.

When analysing the results obtained for VGG16+EfficientNetB7, the Grad-CAM method obtains better results for the IoU parameter and is more stable, contrary to the CAM method. It can be suspected that both networks make different decisions on different assumptions, but the Grad-CAM methods does it better. Note that for CNN + EfficientNetB7 and CNN + ResNet50, the results obtained by both methods are similar and not good. Hence, it is difficult to choose the best one.

In the case of ResNet50 + VGG16, the results obtained by both methods are different, and the Grad-CAM method is better and more stable in selected areas, because it achieves IoU better by 22% and CaD is smaller by 41%.

Based on the results obtained with the use of the Grad-CAM and CAM methods, in general, both methods achieve similar results and indicate similar saliency areas, but the Grad-CAM method on average is slightly better ($\bar{IoU}=0.6753$ and $\bar{CaD}=0.1951)$. As a final remark, it is worth mentioning that there are some architectures for which some differences can be observed. It should be noted that the coverage measured between different methods to determine the saliency areas of the same network architectures is higher than when comparing the results of a given method for different network architectures.

## 4. Conclusions

In this paper we presented the use of deep neural models and methods determining the saliency areas for solving the binary classification problem to determine whether a patient had a brain tumour or not. Of course, the opacity of deep neural networks raises many questions of explainability, which these networks use to make decisions. Saliency maps can identify parts of an image that best represent the decision making of convolutional neural networks. Based on transfer learning, three pre-trained network models were used and tested on the set of brain tumour images from the Kaggle platform. To indicate the desirability of using these models, the results of our simple convolutional neural network implemented without transfer learning were presented. This intended aim was enacted by learning from the beginning. Experiments were carried out to compare the currently available methods of creating the saliency maps in order to check how they solve the problem of interpretability of deep neural network results in the case of medical image analysis. On the basis of the tests, we can conclude that the results obtained using the CoM parameter are consistent with the results obtained using the IoU metric. More complex convolutional neural networks focus on more abstract features, hence the saliency areas are similar.

Future research will be devoted to the performance of other deep networks with the CAM and Grad-CAM methods, solving the problem of multi-class classification of images, with various tumours of the brain. Looking at the results obtained, it is tempting to implement other post hoc explainable AI methods to determine the saliency maps, for example Grad-CAM++ [26], Eigen-CAM [27]. In the study of various brain tumours, AI algorithms that utilize radiomic properties of medical images will be used. In such cases, the importance of each feature in the model’s decision-making process will be evaluated. Furthermore, we would like to use more datasets to make these networks more robust. In addition, to obtain reliable results, medical images used to learn networks should be annotated by utilising the bounding box defined by radiology specialists. However, truly qualitative the extent of this problem remains a challenge because tumours can perfectly disguise themselves.

## Figures and Tables

**Table 1:** Comparison of trained convolutional network architectures.

Architecture	Layers Number	Total Parameters	Trainable Parameters	Train Accuracy	Test Accuracy	Recall	Precision
VGG16	16 + 3	14,978,370	263,682	98.42%	97.88%	98.55%	97.71%
ResNet50	50 + 3	25,947,394	2,359,682	98.99%	97.66%	95.65%	98.17%
EfficientNet	813 + 3	67,047,193	2,949,506	98.37%	98.88%	98.96%	98.40%
CNN	10	97,458	97,458	90.92%	91.96%	95.45%	87.41%

**Table 2:** Comparison of results for the CAM and Grad-CAM methods.

	Intersection over Union (IoU)			Cartesian Distance (CaD) of CoM		
	**CAM**	**Grad-CAM**	$E_{IoU}$ **(%)**	**CAM**	**Grad-CAM**	$E_{CaD}$ **(%)**
CNN + EfficientNet	0.635	0.627	1.26	0.2121	0.2496	−17.72
CNN + VGG	0.447	0.562	−25.73	0.2893	0.2913	−0.69
CNN + ResNet	0.618	0.602	2.59	0.2229	0.2626	−17.78
ResNet + EfficientNet	0.854	0.851	0.35	0.0916	0.1208	−31.92
ResNet + VGG	0.572	0.699	−22.20	0.2396	0.1398	41.65
VGG + EfficientNet	0.579	0.711	−22.80	0.2156	0.1067	50.50
Avr	0.618	0.675		0.2118	0.1951	

## Data Availability

The dataset is from the Kaggle dataset (openly available on https://www.kaggle.com/datasets/preetviradiya/brian-tumor-dataset (accessed on 11 October 2022)).

## Abbreviations

The following abbreviations are used in this manuscript:

CAM	Class Activation Mapping
Grad-CAM	Gradient-based Class Activation Mapping
CNN	Convolutional Neural Network
IoU	Intersection over Union
CoM	Center of Mass
ResNet	Residual Network
VGG	Visual Geometry Group
MRI	Magnetic Resonance Image
CaD	Cartesian Distance of CoM

## References

[1] McFaline-Figueroa J.R., Lee E.Q. (2018). Brain tumors. Am. J. Med..

[2] White B.J., Berg D.J., Kan J.Y., Marino R.A., Itti L., Munoz D.P. (2017). Superior colliculus neurons encode a visual saliency map during free viewing of natural dynamic video. Nat. Commun..

[3] Saporta A., Gui X., Agrawal A., Pareek A., Truong S.Q.H., Nguyen C.D.T., Ngo V.-D., Seekins J., Blankenberg F.G., Ng A.Y. (2022). Benchmarking saliency methods for chest X-ray interpretation. Nat. Mach. Intell..

[4] Wang Z.J. (2021). Probing an AI regression model for hand bone age determination using gradient-based saliency mapping. Sci. Rep..

[5] Ghosh S., D’Angelo G., Glover A., Iacono M., Niebur E., Bartolozzi C. (2022). Event-driven proto-object based saliency in 3D space to attract a robot’s attention. Sci. Rep..

[6] Amorim J.P., Abreu P.H., Santos J., Cortes M., Vila V. (2023). Evaluating the faithfulness of saliency maps in explaining deep learning models using realistic perturbations. Inf. Process. Manag..

[7] Ayhan M.S., Kümmerle L.B., Kühlewein L., Inhoffen W., Aliyeva G., Ziemssen F., Berens P. (2022). Clinical validation of saliency maps for understanding deep neural networks in ophthalmology. Med. Image Anal..

[8] Saeedi S., Rezayi S., Keshavarz H., Kalhori S.R.N. (2023). MRI-based brain tumor detection using convolutional deep learning methods and chosen machine learning techniques. BMC Med. Inform. Decis. Mak..

[9] Humayun M., Khalil M.I., Alwakid G., Jhanjhi N.Z. (2020). Superlative Feature Selection Based Image Classification Using Deep Learning in Medical Imaging. J. Healthc. Eng..

[10] Saida D., Premchand P. (2022). Brain Tumor Identification using Dilated U-Net based CNN. Int. J. Comput. Commun. Control.

[11] Khan M.A., Ashraf I., Alhaisoni M., Damaševicius R., Scherer R., Rehman A., Bukhari S.A.C. (2020). Multimodal brain tumor classification using deep learning and robust feature selection: A machine learning application for radiologists. Diagnostics.

[12] Amin J., Sharif M., Gul N., Yasmin M., Shad S.A. (2020). Brain tumor classification based on DWT fusion of MRI sequences using convolutional neural network. Pattern Recognit. Lett..

[13] Sajjad M., Khan S., Muhammad K., Wu W., Ullah A., Baik S.W. (2019). Multi-grade brain tumor classification using deep CNN with extensive data augmentation. J. Comput. Sci..

[14] Kokkalla S., Kakarla J., Venkateswarlu I.B., Singh M. (2021). Three-class brain tumor classification using deep dense inception residual network. Soft Comput..

[15] Alsubai S., Khan H.U., Alqahtani A., Sha M., Abbas S., Mohammad U.G. (2022). Ensemble deep learning for brain tumor detection. Front. Comput. Neurosci..

[16] Brain Tumor Dataset. https://www.kaggle.com/datasets/preetviradiya/brian-tumor-dataset.

[17] Varga D. (2022). No-Reference Image Quality Assessment with Convolutional Neural Networks and Decision Fusion. Appl. Sci.

[18] Simonyan. K., Zisserman A. Very Deep Convolutional Networks for Large-Scale Image Recognition. Proceedings of the 3rd International Conference on Learning Representations (ICLR).

[19] He K., Zhang X., Ren S., Sun J. Deep Residual Learning for Image Recognition. Proceedings of the IEEE Conference on Computer Vision and Pattern Recognition (CVPR).

[20] Tan M., Le Q.V. EfficientNet: Rethinking Model Scaling for Convolutional Neural Networks. Proceedings of the International Conference on Machine Learning.

[21] Zhou B., Khosla A., Lapedriza A., Oliva A., Torralba A. Learning Deep Features for Discriminative Localization. Proceedings of the 2016 IEEE Conference on Computer Vision and Pattern Recognition (CVPR).

[22] Selvaraju R.R., Cogswell M., Das A., Vedantam R., Parikh D., Batra D. (2020). Grad-CAM: Visual Explanations from Deep Networks via Gradient-Based Localization. Int. J. Comput. Vis..

[23] Zhang L., Shen Y., Li H. (2014). VSI: A visual saliency induced index for perceptual image quality assessment. IEEE Trans. Image Process..

[24] Zhou W., Yue G., Zhang R., Qin Y., Li H. (2023). Reduced-reference quality assessment of point clouds via content-oriented saliency projection. IEEE Signal Process. Lett..

[25] Rezatofighi H., Tsoi N., Gwak J., Sadeghian A., Reid I., Savarese S. Generalized Intersection Over Union: A Metric and a Loss for Bounding Box Regression. Proceedings of the 2019 IEEE/CVF Conference on Computer Vision and Pattern Recognition (CVPR).

[26] Chattopadhay A., Sarkar A., Howlader P., Balasubramanian V.N. Grad-CAM++: Generalized gradient-based visual explanations for deep convolutional networks. In Proceeding of the 2018 IEEE Winter Conference on Applications of Computer Vision (WACV).

[27] Bany Muhammad M., Yeasin M. (2021). Eigen-CAM: Visual explanations for deep convolutional neural networks. SN Comput. Sci..

